# Tumor-associated macrophages in cancer: from mechanisms to application

**DOI:** 10.1186/s43556-025-00396-y

**Published:** 2025-12-19

**Authors:** Wan Tang, Xin Wang, Bing Han, Shu-Heng Jiang, Hongshi Cao

**Affiliations:** 1 The First Hospital of Jilin University, No.71, Xinmin Street, Changchun City, Jilin Province 130021 P.R. China; 2 Departments of Breast Surgery, General Surgery Center, The First Hospital of Jilin University, No.71, Xinmin Street, Changchun City, Jilin Province 130021 P.R. China; 3 State Key Laboratory of Systems Medicine for Cancer, Shanghai Cancer Institute, Ren Ji Hospital, School of Medicine, Shanghai Jiao Tong University, Shanghai, 200240 P.R. China

**Keywords:** Tumor microenvironment, Macrophage polarization, Targeted therapy, Prognostic prediction, Immune infiltration

## Abstract

Tumor-associated macrophages (TAMs) are central constituents of the tumor microenvironment (TME), recruited from circulating monocytes through chemotactic signals, and they execute complex, multifaceted functions throughout tumor progression. Functionally heterogeneous, TAMs are broadly classified into distinct subtypes that display a dynamic duality, capable of shifting between tumor-suppressive and tumor-promoting states, though the pro-tumorigenic functions tend to dominate across multiple cancer types. The polarization of TAMs is modulated by diverse cytokines and signaling networks within the TME. Key pro-tumor mechanisms include activating proliferative signaling pathways, enhancing invasive and metastatic potential, establishing an immunosuppressive TME through immune cell interactions, and conferring therapy resistance. The spatial heterogeneity of TAMs further underscores the predictive relevance. Translational research increasingly focuses on TAM-targeting strategies such as inhibiting recruitment, depleting subsets, or reprogramming function. Emerging approaches, including nanomedicine-based targeting, macrophage-mediated therapies, and novel drug formulations, highlight the importance of combining conventional treatments with immune checkpoint inhibitors (ICIs). Such combinations help overcome therapeutic resistance and improve clinical outcomes. This review systematically summarizes recent advances in TAM biology and plasticity, biomarkers from single-cell and spatial analyses for distinguishing TAM subsets, and their prognostic relevance in immunotherapy. It also discusses TAM-targeting strategies and their synergistic potential with existing therapies. Together, these insights lay the foundation for next-generation cancer treatments that precisely target TAMs to overcome therapy resistance and improve patient survival.

## Introduction

Globally, nearly 20 million new cancer cases were diagnosed in 2022, accompanied by approximately 9.7 million cancer-related deaths [1]. Although recent progress in cancer prevention, early detection, and treatment has led to improved survival outcomes, increasing rates of several cancer types, alongside an aging population, continue to fuel the growing burden of cancer cases and associated deaths. As a result, there is an urgent need for substantial advances in the treatment of solid tumors. The tumor microenvironment (TME) represents a highly complex ecosystem wherein dynamic interactions among malignant cells, stromal constituents, and immune cell populations critically influence disease progression and therapeutic efficacy [2]. Together, these components coordinate hallmark malignant behaviors such as proliferation, invasion, metastatic dissemination, and adaptation to treatment-induced stress [3]. Within the pathologically altered niche, cancer cells engage in bidirectional crosstalk with stromal and immune elements. Key contributors include vasculature that supports angiogenesis, fibroblasts responsible for remodeling the extracellular matrix (ECM), neuroendocrine components, cytokine and chemokine networks, and immune populations spanning both adaptive immunity, such as T cells and B cells, and innate immunity, including neutrophils, mast cells, and tumor-associated macrophages (TAMs) [4]. The intricate web of cellular and molecular interactions fosters a pro-tumorigenic milieu that amplifies tumor aggressiveness.

TAMs constitute not only the most abundant tumor-infiltrating immune population within the TME, but also the predominant innate immune subset. Their critical involvement in tumor initiation, progression, and immune evasion has drawn considerable research interest [5]. Derived from circulating monocytes in peripheral blood, TAMs migrate through the vasculature to tumor sites, where they perform diverse and context-dependent biological functions [6]. The phenotypic polarization and spatial distribution enable TAMs to function as a double-edged sword, capable of either restraining or promoting malignant growth. The abundance and functional state of TAMs are shaped by a variety of factors, such as cytokines, chemotactic cues, and cross-talk with other immune cells [7]. Equally important is the functional plasticity, which allows TAMs to regulate antitumor immunity, angiogenesis, and metastatic dissemination, thereby underscoring the central role in tumor biology and resistance to therapy. Targeting TAMs has become a major focus in cancer immunotherapy. Key strategies include limiting monocyte recruitment, depleting TAM populations, reprogramming the polarization states, and modulating their metabolic activity. While most TAM-directed therapies are still in preclinical development, with efforts centered on optimizing the therapeutic window by balancing efficacy and systemic toxicity, a new generation of approaches offering improved precision and controllability is rapidly emerging. Among the chimeric antigen receptor macrophage (CAR-M) platforms engineered to enable tumor antigen-specific phagocytosis, newly developed therapeutic agents, and nanotechnology-based delivery systems designed to precisely regulate TAM polarization or eliminate immunosuppressive subpopulations [8]. Together, the advances seek to address the spatial heterogeneity of TAM phenotypes and enhance treatment specificity within the dynamically evolving tumor ecosystem. Despite ongoing advances, several areas of TAM biology remain contentious. The conventional antitumoral/pro-tumoral classification, while conceptually useful, oversimplifies the considerable heterogeneity of TAMs. Single-cell RNA sequencing (scRNA-seq) has challenged the binary view, uncovering distinct functional signatures among TAM subpopulations [9–13]. In addition, the prognostic significance of TAMs varies across cancer types, with conflicting evidence often attributed to spatial heterogeneity, such as phenotypic differences between TAMs at the invasive margin and those in the tumor core, or to inconsistencies in marker-based identification methods. Furthermore, the optimal therapeutic approach for targeting TAMs remains an active subject of debate.

This review outlines the developmental origins and heterogeneity of TAMs and examines their contributions to primary tumor growth, metastatic dissemination, immune evasion, and therapy resistance in human solid tumors, particularly triple-negative breast cancer (TNBC). We discuss recent insights into tumor-promoting secretory factors derived from TAMs and analyze the signaling pathways and multi-layered regulatory mechanisms that control TAM functionality, which reveal novel molecular interactions and potential avenues for TAM reprogramming. Key advances in identifying biomarkers that differentiate tumor-promoting from tumor-restraining TAM subsets are highlighted, along with the clinical relevance. An overview of leading TAM-directed therapeutic strategies currently in or approaching clinical evaluation is provided, followed by a critical assessment of the current limitations and prospects. The integration of conventional treatments with precision-targeting approaches that respond to dynamic changes in the TME will foster the development of novel agents and establish a conceptual framework for clinical trials exploring TAMs as promising therapeutic targets in oncology.

## Origin and phenotypic polarization of TAMs

TAMs constitute the most prevalent immune infiltrates in the tumor stroma and display functional duality, capable of mediating both anti-tumor and pro-tumor immune responses. In the early stages of tumor transformation, pro-inflammatory immune cells including monocytes, are recruited to the site by initial inflammatory cues. The signals also activate embryonic tissue-resident macrophages residing in the local microenvironment [14, 15].

Chemokines released by tumor cells play crucial roles in recruiting monocyte-macrophage lineages during tumor progression [16–18], with CCL2 serving a particularly central function [19–22]. CCL2-stimulated macrophages produce VEGF-A and activate the AKT signaling pathway, leading to upregulation of STC1 in melanoma models, which in turn promotes YAP activation and further amplifies CCL2 expression [22]. Specific signaling axes such as PI3K-AKT-SELE/VCAM1 and CCL16-CCR1 have been implicated in TAM recruitment in pancreatic ductal adenocarcinoma and hepatocellular carcinoma (HCC),respectively [23, 24]. TAMs also engage in dynamic crosstalk with other cellular components in the TME, creating self-reinforcing recruitment loops. For instance, pancreatic cancer (PC) cells secrete PGE₂ and tumor necrosis factor (TNF) to attract macrophages; once infiltrated, the macrophages provoke inflammatory reactions in neighboring cancer cells, further amplifying PGE₂ and TNF production via IL-1β signaling and attracting more TAMs [25]. A similar mutually reinforcing relationship exists between tumor-associated neutrophils (TANs) and TAMs. TAN-derived chemokines such as CCL2, CCL5, and CSF1 help recruit TAMs, while TAMs in turn release neutrophil chemoattractants. Notably, factors like CSF1 and CXCL8 are further upregulated in TAM-TAN co-culture systems. Cancer-associated fibroblasts (CAFs) also contribute to the network by promoting TAM recruitment through the CXCL12/CXCR4 axis in breast cancer (BC) and the IL-8/CXCR2 axis in colorectal cancer (CRC) [26].

Naive macrophages (M0) acquire specialized functional phenotypes through a process termed macrophage polarization in response to specific stimuli and signals. The polarization broadly classifies macrophages into two extreme states: classically activated, anti-tumoral macrophages, which are characterized by the release of pro-inflammatory mediators, and alternatively activated, pro-tumoral macrophages, associated with anti-inflammatory cytokine production. The differentiation is directed by distinct cytokine milieus and transcriptional regulatory programs [27]. Anti-tumoral macrophages are typically activated by Th1 signals such as TNF and IFN-γ, and express surface markers including CD80, CD86, and iNOS. They exert anti-tumor effects through the secretion of pro-inflammatory cytokines like TNF and IL-2, generation of reactive oxygen and nitrogen species, and enhanced antigen presentation to T cells. As such, anti-tumoral macrophages are critical for initiating anti-tumor immunity in most cancer types. In contrast, pro-tumoral macrophages polarization is driven by IL-4, IL-10, IL-13, and other Th2 cytokines, and is associated with markers such as CD206, arginase 1, and CD163. Through angiogenesis, ECM remodeling, and diverse immunosuppressive mechanisms, pro-inflammatory macrophages facilitate tumor progression and dampen anti-tumor immune responses.

The binary classification originally emerged from in vitro studies of macrophage responses to type 1 or type 2 cytokines [28, 29]. Advances in technology and macrophage biology, however, have since revealed the high degree of plasticity and heterogeneity, enabling dynamic phenotypic adaptation to diverse microenvironmental cues. New evidence further indicates that anti-tumoral like macrophages also foster tumor development by secreting inflammatory mediators that shape a pro-tumorigenic TME. For example, anti-tumoral macrophages have been shown to promote cancer stemness and immune evasion in oral squamous cell carcinoma (OSCC), glioma, BC, and HCC [30–33]. On the other hand, owing to the anti-inflammatory and tissue-repair functions, pro-tumoral macrophages also exert context-dependent beneficial effects in cancer therapy [34]. Therefore, categorizing TAMs solely as anti-tumoral or pro-tumoral represents a substantial oversimplification [35].

scRNA-seq has identified novel TAM subpopulations that contribute critically to tumor growth and proliferation. In HCC, THBS1⁺ macrophages (myeloid-derived suppressor cells-like macrophages) and C1QA⁺ macrophages (TAM-like macrophages) are enriched in tumor tissues [36]. The subsets share features with conventional TAMs yet display greater complexity than the classical anti-tumoral/pro-tumoral model, supporting the classification as distinct subtypes. A newly identified FABP4⁺C1q⁺ macrophage subtype has also been described, specialized in enhancing cytokine production, inflammatory and chemotactic responses, neutrophil activation, leukocyte migration, pro-inflammatory secretion, phagocytosis, and anti-apoptotic activity [37]. The mechanisms collectively underlie its anti-tumor function. In melanoma, a MerTK⁺ macrophage subpopulation exhibits potent immunosuppressive activity that supports tumor growth [38]. In glioma, a macrophage subtype defined by high FN1 expression, termed FN1⁺ TAMs, has been shown to critically promote tumor recurrence [29]. Together, these findings underscore the extensive heterogeneity and functional complexity of TAMs. Deeper investigation of the newly defined macrophage subtypes unveils novel targets for cancer therapy.

## Mechanisms of TAMs in tumor progression

TAMs are aberrantly accumulated in tumors and act as pivotal drivers of disease progression through multifaceted crosstalk mediated by cytokines and growth factors. Soluble tumor-derived signals orchestrate the recruitment of monocytes from peripheral blood, which then undergo polarization into pro-tumoral TAMs within the immunosuppressive TME. Through diverse signaling pathways, the reprogrammed TAMs engage with cancer cells to sustain their survival and proliferation, enhance migratory capacity necessary for tissue invasion and intravasation, dampen antitumor immunity, and foster therapy resistance [39].

### TAMs promoting tumor growth and proliferation

**Fig. 1:**
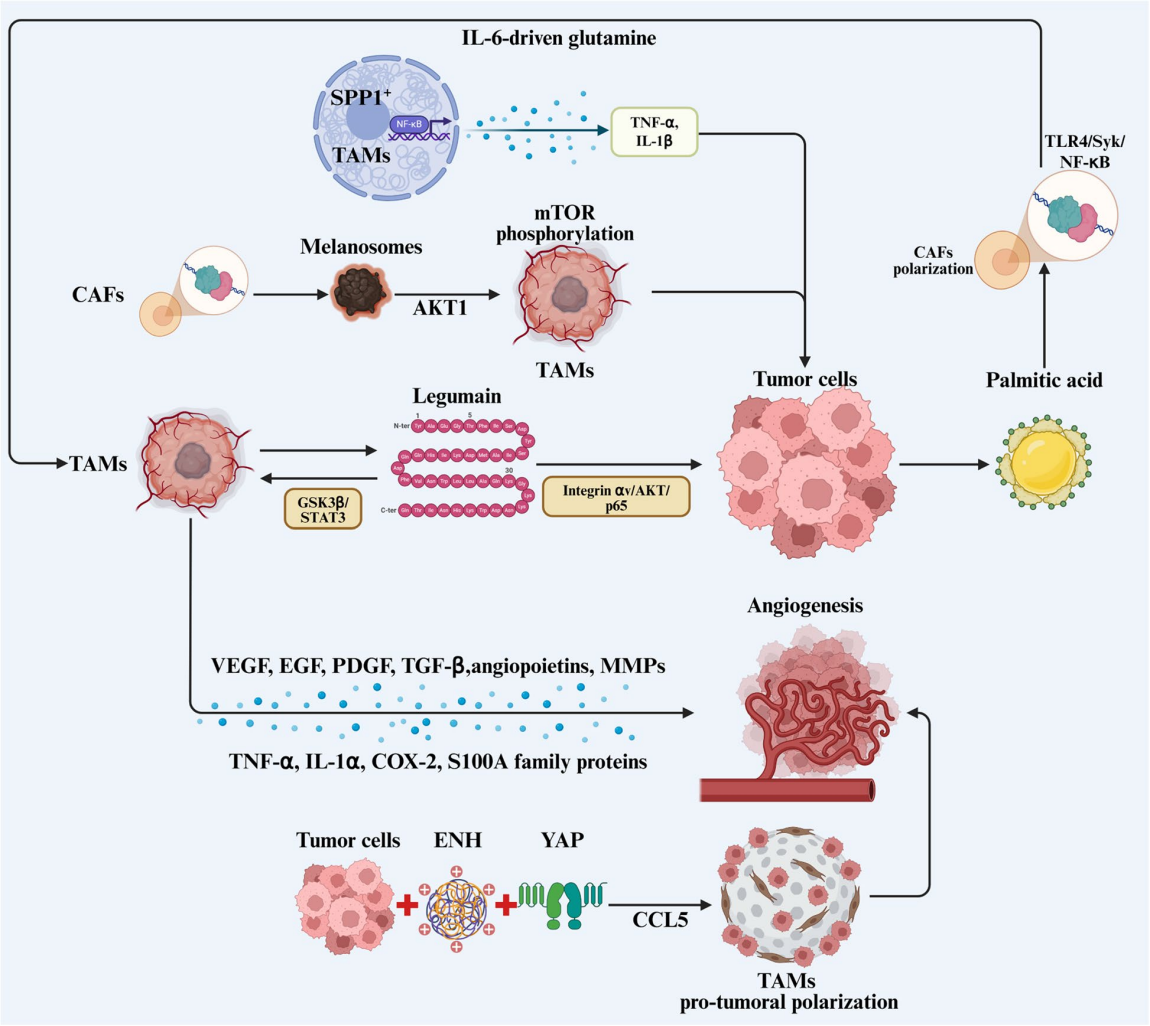
TAMs critically support tumor growth and proliferation through multiple mechanisms. They secrete a broad spectrum of cytokines and chemokines that provide direct mitogenic signals to cancer cells. Additionally, TAMs facilitate tumor expansion by remodeling the ECM and promoting angiogenesis, thereby supplying essential structural support and nutrient provision to the TME. Abbreviations: TAMs, tumor-associated macrophages; CAFs, cancer-associated fibroblasts

TAMs play a critically recognized role in promoting tumor growth and proliferation, largely mediated through the abundant secretion of cytokines and chemokines, as established in both preclinical and clinical settings (Fig. 1). TAM-derived legumain exerts dual regulatory roles in glioblastoma (GBM) by promoting macrophage infiltration in an autocrine manner via the GSK3β/STAT3 pathway, while in a paracrine fashion, TAM-secreted legumain drives GBM cell proliferation and survival through integrin αv/AKT/p65 signaling [40]. Moreover, SPP1⁺ macrophages augment the secretion of TNF-α and IL-1β via the NF-κB pathway, further supporting proliferation in head and neck squamous cell carcinoma (HNSCC) [41].

Inflammatory processes often drive the formation of dense, fibrotic stroma rich in ECM within tumors. CAFs represent the predominant cellular component of the ECM, typically arising from tissue-resident fibroblasts and other native cells through diverse mechanisms [42]. Notably, TAMs are frequently enriched at the periphery of CAF-dense regions, reflecting active intercellular crosstalk [43]. CAFs facilitate TAM recruitment into the TME and promote the polarization toward a pro-tumoral phenotype through the secretion of various regulatory molecules, thereby amplifying TAM-mediated immunosuppression [44, 45]. Tumor-derived palmitic acid activates a Toll-like receptor (TLR)4/Syk/NF-κB signaling cascade in fibroblasts, inducing inflammatory CAF polarization and IL-6-driven glutamine synthesis, which in turn promotes TAM polarization and supports tumor growth by modulating TAM composition [46]. In vivo co-transplantation of macrophages with fibroblast-derived melanosomes further enhances tumor growth, proliferation, and angiogenesis [47]. The process involves AKT1 transfer from melanosomes to macrophages, activating mTOR phosphorylation and triggering excessive VEGF secretion, which elevates proliferation rates in melanoma cells.

Orderly vascular and lymphatic networks help establish a nutrient-rich, immune-privileged microenvironment that facilitates tumor progression [48]. As central regulators of tumor angiogenesis, TAMs secrete key pro-angiogenic factors, such as VEGF, EGF, PDGF, TGF-β, and angiopoietins, and facilitate perivascular ECM breakdown via matrix metalloproteinase release [49]. They also indirectly promote angiogenesis through molecules like TNF-α, IL-1α, and COX-2, which activate pro-angiogenic programs in tumor cells [50]. Additional "non-classical" mediators from TAMs including growth factors, enzymes, and ECM proteins, further fine-tune angiogenic regulation [51]. For instance, S100A family proteins from TAMs stimulate endothelial cell proliferation, migration, and tube formation in vitro and in vivo [52, 53]. scRNA-seq in CRC has identified an SPP1⁺ TAM subset enriched in gene sets associated with angiogenesis, ECM-receptor interaction, and vascular development [54, 55]. In BC, LYVE1⁺ perivascular TAMs localize adjacent to αSMA⁺ pericyte-like mesenchymal cells [56] The perivascular TAMs organize into multicellular clusters through an IL-6/CCR5-mediated mechanism [57]. In lung adenocarcinoma (LUAD), high ENH expression correlates with increased microvessel density and TAM infiltration. ENH promotes YAP nuclear translocation and CCL5 transcription, augmenting monocyte chemotaxis, TAM accumulation, pro-tumoral polarization, and ultimately fueling LUAD angiogenesis and growth [58]. Genetic ablation of TSC1, a negative regulator of mTORC1 in TAMs, inhibits tumor growth independent of adaptive immunity. Whereas wild-type TAMs display pro-inflammatory and pro-angiogenic transcriptional profiles, TSC1-deficient TAMs vacate perivascular niches, reduce Procr⁺ endothelial progenitor populations, and normalize vascular permeability, collectively inducing tumor hypoxia and cancer cell death [59].

As integral components of the TME, TAMs vigorously support tumor growth and proliferation via multiple synergistic mechanisms. Their functional contributions begin with recruitment into tumors by tumor-derived factors and subsequent polarization into pro-tumoral-like, pro-tumoral phenotypes within the immunosuppressive TME. The activated TAMs secrete a spectrum of growth factors that directly stimulate cancer cell proliferation, while concurrently supporting tumor expansion through ECM remodeling and angiogenesis, ensuring both structural reinforcement and metabolic supply.

### TAMs facilitating tumor invasion and metastasis

**Fig. 2:**
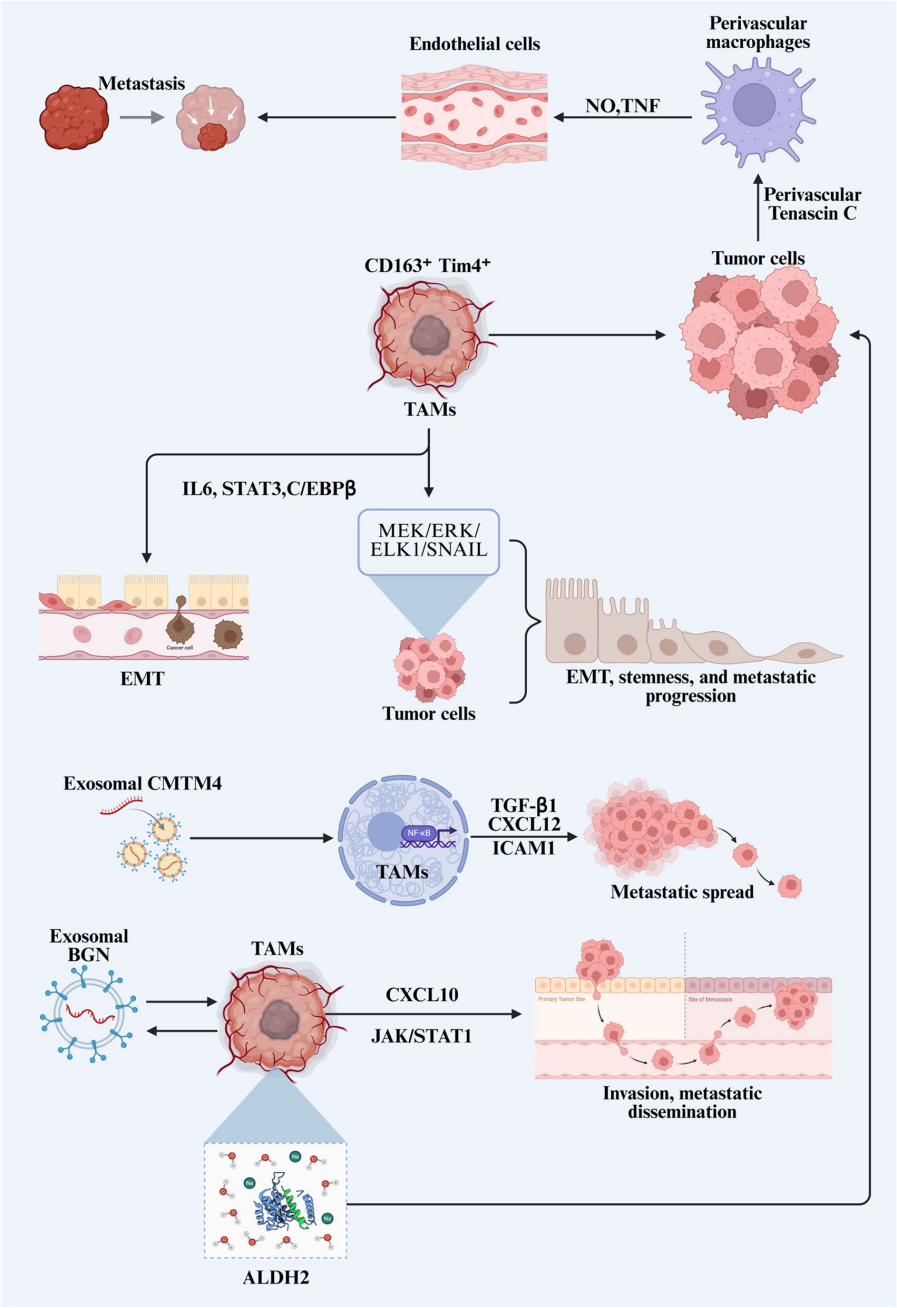
TAMs are master regulators of tumor invasion and metastasis. TAMs facilitate local tissue invasion, intravasation into the circulation, and eventual metastatic outgrowth at distant organs, thereby coordinating multiple steps of the metastatic cascade. Abbreviations: TAMs, tumor-associated macrophages; EMT, epithelial mesenchymal transition

The metastatic dissemination of cancer cells initiates with the invasion into the bloodstream [60]. The invasive process comprises multiple steps: detachment from surrounding tissue architecture, epithelial-mesenchymal transition (EMT), degradation of the ECM, and increased cellular motility [61]. TAMs exert regulatory control over all stages of invasion and metastasis (Fig. 2), thereby contributing to unfavorable patient outcomes [62].

TAMs play an instrumental role in establishing pre-metastatic niches [63–65]. They are recruited to the sites by various tumor-derived factors including CCL2, CSF-1, VEGF, TNF-α, and TGF-β, where they subsequently enhance cancer cell survival [64]. In murine models of ovarian cancer (OC), resident CD163⁺Tim4⁺ macrophages located in the omentum have been demonstrated to facilitate metastatic dissemination [66]. During BC metastasis to the lungs, functional interplay between endothelial cells and perivascular macrophages promotes the formation of a specialized vascular niche [67]. Mechanistically, perivascular Tenascin C produced by tumor cells activates TLR4-dependent perivascular macrophages within pre-metastatic niches, resulting in upregulated expression of NO and TNF to induce endothelial cells-mediated production of niche components. The molecular changes have been functionally linked to enhanced lung metastatic colonization in vivo.

TAM-secreted cytokines such as IL-1β, IL-8, TNF-α, and TGF-β promote EMT in cancer cells [68], leading to disrupted intercellular junctions and accelerated tumor cell movement. To facilitate ECM breakdown, TAMs release several proteolytic enzymes including cathepsins, matrix metalloproteinases, and serine proteases [61, 69]. In gallbladder carcinoma, pro-tumoral-like TAMs activate the MEK/ERK/ELK1/SNAIL pathway in cancer cells, thereby promoting EMT, stemness, and metastatic progression [70]. In LUAD, TAMs sustain IL-6 expression through a positive feedback circuit involving IL6, STAT3, and C/EBPβ. The released IL-6 activates EMT signaling in cancer cells, enhancing their migratory and invasive capabilities [71]. In OC, exosomal CMTM4 activates the NF-κB pathway in TAMs, increasing secretion of TGF-β1 and CXCL12 to suppress immune responses while simultaneously upregulating intercellular adhesion molecule-1 expression. The cascade further reinforces pro-tumoral polarization and promotes metastatic spread [72]. Similarly, gastric cancer (GC)-derived exosomal BGN is internalized by macrophages, where it interacts with NONO protein to drive pro-tumoral polarization and CXCL10 upregulation. The activation of the JAK/STAT1 signaling pathway subsequently enhances GC cell proliferation, invasion, and metastatic dissemination [73]. Metabolic reprogramming also significantly influences tumor metastasis, with increased acetyl-CoA generation representing a characteristic metabolic adaptation in metastatic cancers. Activation of the lipid peroxidation-aldehyde dehydrogenase 2 axis in TAMs stimulates acetate production and secretion, elevating acetate availability for HCC cells and consequently promoting pulmonary metastasis [74].

After EMT induction and stromal reorganization, tumor cells proceed to intravasate into blood vessels, a step facilitated by a paracrine signaling loop involving tumor-derived CSF1 and macrophage-secreted EGF that directs tumor cell migration toward vasculature [39, 60, 75]. Tumor cell-induced platelet aggregation promotes TAM recruitment to lung metastases through platelet-derived chemokines conditioned by B16 cells. In tumor cell-induced platelet aggregation models, the recruited TAMs undergo pro-tumoral repolarization by B16 cells and display elevated PD-L1 expression, ultimately enabling tumor cells to evade immune detection and accelerating hematogenous spread in malignant melanoma [76]. The inherent mobility and abundance of myeloid cells enable tumor cells associated with TAMs to acquire heightened metastatic competence and increased propensity for distant dissemination. Spatial transcriptomic analyses have revealed that NRF2-activated myeloid cells exhibiting pro-tumor TAM characteristics preferentially accumulate in hemorrhagic tumor regions [77]. Heme, an erythrocyte metabolite, serves as a crucial microenvironmental cue that directs macrophages toward pro-tumor functions. NRF2 amplifies intracellular heme signaling to generate pro-tumor TAMs that stabilize EMT and augment cancer invasiveness and metastatic potential.

In conclusion, TAMs function as master regulators of tumor invasion and metastasis, enabling tumor cells to circumvent immune surveillance, invade adjacent tissues, enter the circulation through diverse mechanisms, and successfully colonize distant organs to establish metastatic lesions.

### TAMs suppressing anti-tumor immune responses

**Fig. 3:**
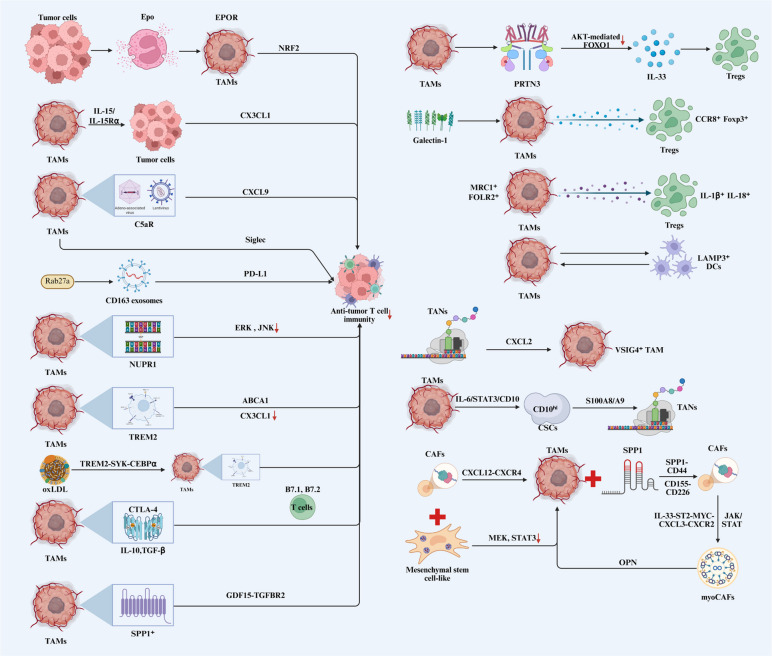
TAMs orchestrate tumor immune escape by dynamically remodeling the tumor immune microenvironment. Through direct interactions and communication with various stromal and immune cells, including T cells, DCs, and CAFs, TAMs establish an immunosuppressive landscape that suppresses adaptive immunity and facilitates immune evasion. Abbreviations: TAMs, tumor-associated macrophages; Tregs, regulatory T cells; DCs, dendritic cells; CAFs, cancer-associated fibroblasts; TANs, tumor-associated neutrophils

Despite the capacity of the innate and adaptive immune systems to restrain tumor formation and progression, cancer cells evade immune surveillance through multiple mechanisms. As key constituents of the TME, TAMs undermine adaptive immune responses by interacting with immune cells, such as T cells, dendritic cells (DCs), and TANs, and fibroblasts, thereby facilitating tumor immune evasion (Fig. 3).

#### TAMs and T cells

T lymphocytes play a central role in adaptive immunity [43], with direct interactions between TAMs and T cells being critically involved in tumor-associated immunosuppression. TAMs restrict CD8⁺ T cell function in breast cancer through collagen deposition and metabolic reprogramming of the TME [7]. Tumor-derived erythropoietin engages its receptor EPOR on TAMs to autonomously establish a non-inflammatory TME in HCC [78]. EPO signaling induces TAM immunomodulation through NRF2-mediated heme depletion, resulting in compromised anti-tumor T cell immunity. TAMs also indirectly regulate T cell function; the IL-15/IL-15Rα complex has been shown to inhibit breast cancer cell secretion of CX3CL1, thereby limiting T cell infiltration and activation [79]. In OC, elevated C5aR expression on TAMs drives the polarization toward an immunosuppressive phenotype [80]. Genetic deletion or pharmacological blockade of C5aR reprograms macrophages from pro-tumor to anti-tumor states, accompanied by upregulated immune response pathways. The reprogramming enhances cytotoxic T cell-mediated anti-tumor responses in a CXCL9-dependent manner. In GBM and high-grade serous ovarian carcinoma, TAMs suppress T cell infiltration and activation through Siglec-9-dependent mechanisms [81, 82].

TAMs frequently express PD-L1, which engages PD-1 on T cells to inhibit T cell activation and function [83]. In a mouse melanoma model, Rab27a-activated TAM-derived CD163 exosomes carried elevated PD-L1 levels and specifically interacted with stimulated CD8⁺ T cells, suppressing the proliferation and cytotoxic activity within tumors [84]. NUPR1 inhibits ERK and JNK signaling to establish an immunosuppressive environment conducive to tumor progression in HCC [85]. NUPR1 upregulation correlates with enhanced pro-tumoral macrophage polarization and increased expression of PD-L1 and SIRPα immune checkpoints, leading to CD8⁺ T cell exhaustion and reduced immunotherapy responsiveness. TAMs also express the PD-1 receptor. PD-1 expression on TAMs diminishes their phagocytic capacity against tumor cells [86]. Myeloid-specific PD-1 ablation more effectively reduces tumor growth and expands T effector memory populations compared to T cell-specific PD-1 deletion. Similarly, eliminating SHP-2, a regulator of PD-1 signaling in myeloid cells, produces greater tumor growth suppression than its deletion in T cells [87].

Triggering receptor expressed on myeloid cells-2 (TREM2) is expressed on TAMs and promotes immunosuppressive microenvironment formation and tumorigenesis in lung cancer, highlighting its potential as an immunotherapy target [88]. Mechanistically, TREM2 deficiency downregulates ATP-binding cassette transporter A1, causing cholesterol accumulation in TAMs and fostering a pro-inflammatory phenotype. The increases macrophage secretion of CX3CL1, recruiting CD4⁺ T cells and NK cells to tumor sites and enhancing anti-tumor responses. Similar mechanisms operate in HCC, where metabolically adapted cancer cells promote oxidized LDL production [89]. Oxidized LDL polarizes TREM2⁺ TAMs via the TREM2-SYK-CEBPα axis, enabling the TAMs to enhance cancer cell invasion, cytokine resistance, and CD8⁺ T cell dysfunction. CTLA-4, an immune checkpoint molecule expressed on tumor and immune cells, inhibits T cell activation and proliferation. In HNSCC, TAM-expressed CTLA-4 binds B7.1 and B7.2 on T cells, suppressing T cell activation and proliferation [90]. TAMs also secrete immunosuppressive molecules including IL-10 and TGF-β that synergize with CTLA-4 signaling to enhance immunosuppression. In metastatic GC, SPP1⁺ TAMs interact with CD8⁺ exhausted T cells through GDF15-TGFBR2 signaling [91].

Similar to TAMs, regulatory T cells (Tregs) are essential for maintaining immunosuppressive microenvironments and promoting tumor immune evasion. TAMs and Tregs cooperate to enhance immunosuppression and mediate resistance to immune checkpoint inhibitors (ICIs) [92]. TAMs secrete various chemokines to recruit Treg cells [93]. TAM-derived IL-23 stabilizes Tregs and enhances their suppression of cytotoxic T cells, facilitating immune evasion [94]. Macrophage PRTN3 upregulates IL-33 expression in TAMs by inhibiting AKT-mediated FOXO1 ubiquitination and degradation, promoting IL-33-induced Treg differentiation in LUAD [95]. The enhanced TAM-Treg crosstalk compromises anti-tumor immunity. In HCC, galectin-1-induced TAMs recruit CCR8⁺Foxp3⁺ Tregs that reduce CD8⁺ T cell infiltration and promote dysfunction, accelerating tumor progression [96]. Reciprocally, recruited Tregs amplify TAM-mediated immunosuppression. In clear cell renal cell carcinoma, IL-1β⁺ and IL-18⁺ Tregs co-localize with MRC1⁺FOLR2⁺ TAMs at tumor-normal interfaces, forming a positive feedback loop that sustains synergistic pro-tumor effects [97]. Collectively, the interactions establish a self-reinforcing immunosuppressive circuit between TAMs and Tregs within the TME.

#### TAMs and DCs

DCs are specialized antigen-presenting cells that form a crucial link between innate and adaptive immunity, coordinating both immune tolerance and activation [98]. TAMs disrupt the function by secreting inhibitory cytokines that suppress DC maturation and impair antigen presentation. Recent scRNA-seq studies have elucidated the complex interactions between TAMs and DCs within the TME. In hypopharyngeal squamous cell carcinoma, scRNA-seq analysis demonstrated cooperative interactions between TAMs and LAMP3⁺ DCs that establish an immunosuppressive milieu favoring tumor progression [99]. The finding highlights the sophisticated crosstalk between TAMs and DCs in modulating anti-tumor immunity and driving disease progression.

#### TAMs and TANs

TANs recruit macrophages to the TME through CXCL2 secretion and influence the polarization state [100]. A recently identified VSIG4⁺ TAM subset exhibits strong immunosuppressive properties in aggressive cancers, anaplastic thyroid cancer and PC [101]. Genetic ablation of VSIG4 in TAMs significantly reduces lactate production and histone H3 lysine 18 lactylation, resulting in diminished STAT3-mediated SPP1 transcription and disrupted TAN-TAM communication. Additionally, TAMs promote allogeneic CD10ʰⁱ cancer stem cells (CSC) through IL-6/STAT3/CD10 signaling [102]. In OSCC, the CD10ʰⁱ CSCs subsequently recruit and reprogram TANs into an immunosuppressive state via S100A8/A9 secretion, revealing a potential therapeutic target for OSCC treatment.

#### TAMs and CAFs

CAFs promote PD-1 upregulation on pro-tumoral-type TAMs, correlating with suppressed innate and adaptive anti-tumor immunity [86]. In TNBC, monocytes recruited via the CAF-driven CXCL12-CXCR4 axis acquire pro-tumor lipid-associated macrophage characteristics that sustain immunosuppression [26]. Combined MEK and STAT3 inhibition reprograms pro-tumoral-like macrophages toward an anti-tumoral phenotype by conferring mesenchymal stem cell-like attributes to CAFs, overcoming therapeutic resistance in PC [103]. In OSCC, TAMs with specific SPP1 expression activate fibroblasts through SPP1-CD44 and CD155-CD226 interactions, remodeling metastatic lymph nodes to support tumor cell colonization [104]. Integrated single-cell and spatial transcriptomic analyses have identified organized spatial networks of SPP1⁺ macrophages and FAP⁺ CAFs in solid malignancies [45, 105, 106]. CAF-to-myoCAF differentiation in PC occurs through IL-33-ST2-MYC-CXCL3-CXCR2 signaling or JAK/STAT activation by macrophage-derived progranulin and cancer cell-secreted leukemia inhibitory factor. Conversely, myoCAFs facilitate metastasis and secrete osteopontin to reinforce immunosuppressive macrophage phenotypes and T cell dysfunction [107, 108].

TAMs are pivotal regulators of immune escape, dynamically remodeling the tumor immune landscape. They fulfill a dual role as immunosuppressors and tumor promoters by driving tumorigenesis and establishing an immunosuppressive TME. Consequently, reprogramming macrophage-mediated crosstalk represents a compelling therapeutic strategy.

### TAMs and drug resistance in tumors

**Fig. 4:**
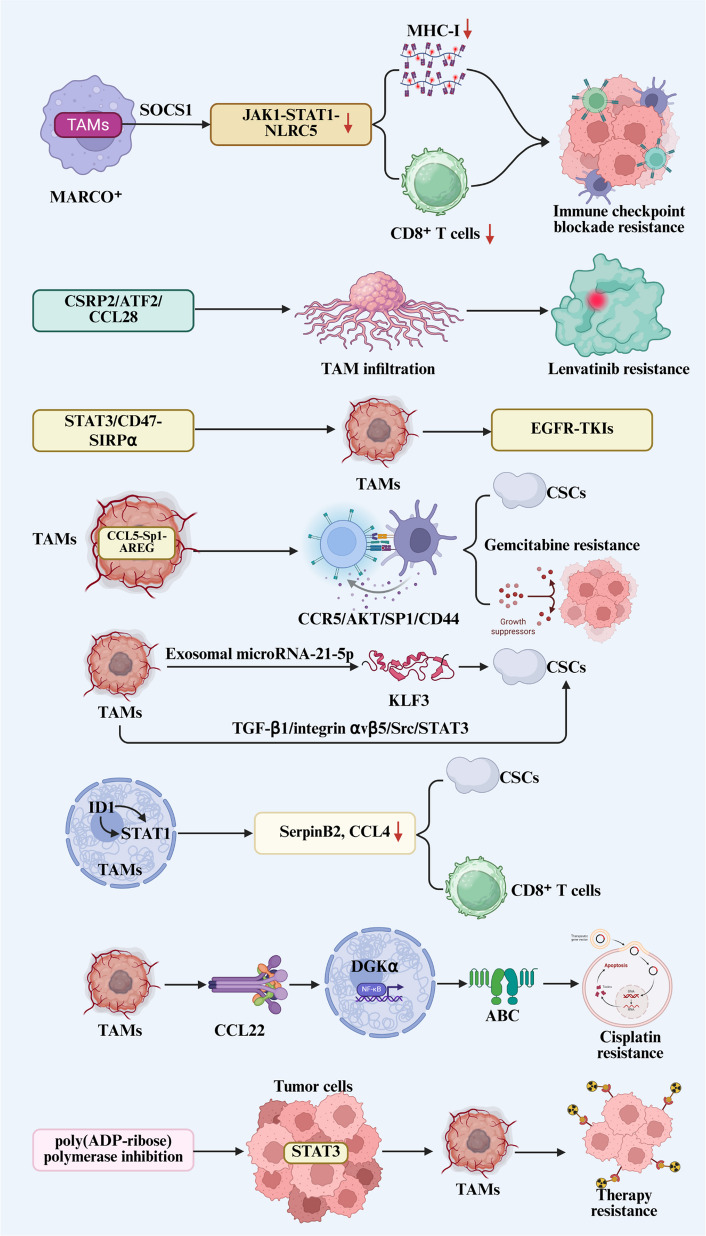
TAMs play a central role in mediating resistance to multiple cancer therapies. Their inherent resilience and capacity to impair the efficacy of chemotherapy, radiotherapy, and immunotherapy are reinforced by positive feedback mechanisms, creating a robust barrier to successful treatment. Abbreviations: TAMs, tumor-associated macrophages; CSCs, cancer stem cells; ABC, ATP-binding cassette

TAMs display inherent resistance to multiple forms of therapy and actively compromise the efficacy of diverse treatment modalities, including immunotherapy, chemotherapy, radiotherapy, and other interventions [109] (Fig. 4). Infiltrating MARCO⁺ TAMs upregulate SOCS1 expression, thereby impeding JAK1 kinase activity [110]. The inhibition disrupts the JAK1-STAT1-NLRC5 signaling cascade, resulting in downregulated MHC-I expression and diminished cytotoxicity of CD8⁺ T cells, a key mechanism responsible for immune checkpoint blockade resistance in renal cell carcinoma. CSRP2 promotes extensive TAM infiltration and modulates the TME through the CSRP2/ATF2/CCL28 axis to drive HCC progression and confer lenvatinib resistance [111]. Moreover, TAM reprogramming via the STAT3/CD47-SIRPα axis facilitates acquired resistance to EGFR-tyrosine kinase inhibitors (TKIs) in lung cancer [112].

CSCs, also known as tumor-initiating or tumor-maintaining cells, constitute a stem-like subpopulation within tumors that demonstrates marked resistance to chemotherapy and radiotherapy [113]. TAMs contribute to CSC maintenance by supporting stemness properties and establishing a specialized niche that promotes CSC survival and drug tolerance [114]. A macrophage-CCL5-Sp1-AREG feedback circuit enhances stemness in PC cells and drives gemcitabine resistance through CCR5/AKT/SP1/CD44 pathway activation [115]. Additionally, pro-tumoral macrophage-derived exosomal microRNA-21-5p promotes pancreatic CSC differentiation and activity by targeting the transcriptional repressor KLF3 [116]. In CRC, ID1 expression in TAMs facilitates interaction with STAT1, leading to its cytoplasmic sequestration and suppression of STAT1-dependent transcription of SerpinB2 and CCL4, both secreted factors involved in CSC inhibition and CD8⁺ T cell recruitment [117]. Reduced ID1 expression correlates with improved CRC prognosis and heightened sensitivity to immunotherapy and chemotherapy. In GBM, TAMs directly sustain tumor stemness via the TGF-β1/integrin αvβ5/Src/STAT3 pathway [118]. Collectively, the observations indicate that TAMs sustain CSC stemness and reinforce therapy resistance primarily through secretion of effector molecules such as chemokines, cytokines, proteins, and miRNAs. Interfering with TAM recruitment or immunosuppressive activity thus represents a promising approach to disrupt CSCs and sensitize tumors to chemotherapy and immunotherapy.

Beyond CSC interactions, TAMs augment tumor cell resistance to chemotherapy and targeted agents through secretion of anti-apoptotic factors and modulation of cell cycle dynamics. In esophageal squamous cell carcinoma (ESCC), stromal TAM-derived CCL22 activates the DGKα/NF-κB pathway, upregulating multiple ATP-binding cassette transporters and reducing intracellular cisplatin accumulation, thereby promoting cisplatin resistance [119]. Consequently, TAM-mediated mechanisms represent a major contributor to chemotherapy failure [120]. Although poly(ADP-ribose) polymerase inhibition shows efficacy in BRCA-mutated OC, it concurrently upregulates STAT3 signaling in tumor cells, inducing pro-tumor TAM polarization and ultimately driving therapy resistance [121].

TAMs promote broad-spectrum therapy resistance in cancer through the multifaceted mechanisms outlined above. The processes are frequently amplified by positive feedback mechanisms. The insights not only clarify how TAM targeting restores treatment sensitivity, but also emphasizes the clinical potential of combining therapies against TAM-derived resistance drivers with conventional treatments, suggesting novel combination strategies to overcome therapeutic resistance in oncology.

## Therapeutic potential of targeting TAMs

Macrophages exhibit remarkable phenotypic plasticity in response to dynamic signals within the TME, allowing them to perform dual roles in both inhibiting and promoting tumor growth. Current therapeutic strategies directed against TAMs encompass multiple approaches: disrupting their recruitment, depleting existing macrophage populations, inhibiting key signaling pathways to suppress pro-tumoral-like polarization, reprogramming TAMs toward antitumor anti-tumoral-like phenotypes, implementing CAR-M therapy, and modulating metabolic processes. The strategies represent promising avenues for targeting TAMs in malignant diseases. We further review clinical evidence on combination therapies and novel pharmaceutical agents, providing substantial support for these innovative approaches to suppress tumor growth and progression.

### Strategies for targeting TAMs

The overall framework for targeting TAMs centers on two primary objectives: first, controlling TAM numbers by limiting their recruitment and eliminating established populations; and second, modifying TAM phenotype by reprogramming pro-tumoral-type macrophages toward anti-tumoral-like states, thereby reducing their tumor-promoting capabilities.

#### Inhibiting TAM recruitment

Following tumor development, TAMs contribute to establishing an inflammatory milieu that facilitates tumor cell proliferation. Simultaneously, tumor cells secrete various cytokines that recruit additional TAMs through specific signaling pathways, further shaping a TME favorable for cancer progression [121]. Consequently, interrupting macrophage recruitment to tumor tissues disrupts critical signals for macrophage growth and differentiation, ultimately compromising the ability of TAMs to support tumor development and dissemination.

Among the most promising initial targets for limiting TAM differentiation and accumulation is CSF1R [28]. Inhibition of the CSF-1/CSF-1R signaling pathway has proven effective in preventing TAM recruitment [122]. TAMs adversely influence treatment outcomes by modulating tumor-stromal interactions. However, CSF-1/CSF1R blockade also impacts Tregs and DCs within the TME, potentially triggering compensatory mechanisms that activate alternative survival pathways and yield limited clinical benefits [123].

Blocking chemokine signaling constitutes another crucial strategy for inhibiting TAM recruitment, with the CCL2/CCR2 axis emerging as a prominent therapeutic target. CCL2-neutralizing antibodies and CCR2 antagonists such as PF-04136309, RS504393, and CCX872 have been utilized to disrupt CCL2/CCR2 signaling, reduce TAM infiltration into the TME, and enhance the antitumor efficacy of ICIs in preclinical models [124–127]. The Stabilin-1-interacting chitinase-like protein impairs the cytoskeletal response to CCL2, alters TME cellular composition, and ultimately prevents cytokine-mediated TAM recruitment [128].

#### Depleting TAMs

Direct removal of TAMs from the TME offers a straightforward approach to counteract the immunosuppression. Zoledronic acid, which undergoes active uptake by macrophages, induces substantial TAM depletion. Combination therapy of zoledronic acid with thymosin α1 or anti-PD-L1 has been demonstrated to significantly alleviate immunosuppression in prostate and liver cancer models, stimulate pro-inflammatory macrophage responses, and activate cytotoxic T cells [129, 130]. Trabectedin, an anticancer chemotherapeutic, selectively eliminates circulating monocytes and tumor-resident TAMs through a TRAIL-dependent mechanism [131]. Preclinical studies in prostate and OC models have reported TAM-mediated antitumor effects of trabectedin [132, 133].

#### Functional reprogramming of TAMs

The functional duality and remarkable plasticity of TAMs offer compelling rationale for targeted anticancer approaches. Reprogramming TAMs represents a promising anticancer strategy; this involves blocking the pro-tumorigenic functions while simultaneously activating antitumor mechanisms [28, 134].

CSF-1/CSF1R-mediated signaling plays a fundamental role in pro-tumoral polarization [135]. In a murine model, BLZ945 effectively suppressed the polarization toward the pro-tumoral phenotype, consequently impeding tumor progression [136]. Another CSF1R inhibitor, pexidartinib, demonstrates comparable activity by selectively disrupting pro-tumoral macrophage function and polarization. This enhances cytotoxic CD8⁺ T cell infiltration and fosters a tumor-suppressive microenvironment through interruption of tumor-pro-tumoral TAM crosstalk, while preserving antitumor functions of anti-tumoral TAMs [137, 138].

Cytokine-directed intervention provides an alternative strategy for modulating TAM polarization and expanding immunotherapeutic possibilities. The CCL2/CCR2 axis serves as a key regulator of TAM biology. The intergenic noncoding RNA LINC00330 specifically interacts with CCL2 and inhibits CCL2/CCR2 autocrine signaling and downstream effectors [139]. The molecular interaction facilitates TAM reprogramming by redirecting polarization from pro-tumoral to anti-tumoral phenotypes. IL-6 drives pro-tumoral-like polarization through JAK/STAT signaling. IL-6 blockade not only induces immunogenic cell death in tumor cells but also reprograms TAMs toward an anti-tumoral phenotype, effectively reversing resistance to ICIs while minimizing immune-related adverse events [140, 141]. MKRN2 functions as an E3 ubiquitin ligase that directly targets NF-κB p65 for proteasomal degradation, thereby constraining NF-κB/COX2-mediated inflammatory signaling [142]. MKRN2 deficiency promotes the transition of TAM polarization from anti-tumoral to protumor pro-tumoral states, establishing it as a critical regulator of immunosuppressive TME formation.

TAMs contribute to antibody-dependent cellular phagocytosis through phagocytic pathway mediation and clearance of antibody-opsonized target cells [143]. Consequently, therapeutic strategies targeting phagocytic checkpoints, enhancing antibody-dependent cellular phagocytosis, or activating macrophages are under active investigation to improve antitumor efficacy. Tumor cells evade phagocytosis by overexpressing CD47, which engages SIRPα on macrophages to transmit a "do not eat me" signal [144–146]. Thus, the CD47-SIRPα axis represents a macrophage immune checkpoint and promising immunotherapeutic target. Disruption of CD47-SIRPα signaling restores phagocytic function by repolarizing TAMs toward an antitumor state, diminishing pro-tumoral-type TAMs while enriching the anti-tumoral phenotype. CD47 inhibition with specific antibodies enhances TAM phagocytic capacity [147]. HCB101, a third-generation CD47-SIRPα inhibitor, binds CD47 with high affinity, potently stimulates macrophage-mediated tumor cell phagocytosis without erythrocyte toxicity, increases the anti-tumoral/pro-tumoral macrophage ratio in the TME indicating pro-inflammatory repolarization, and significantly suppresses tumor growth [148]. Trogocytosis represents another relevant biological process affecting antibody-opsonized tumor cells. Distinct from conventional phagocytosis, trogocytosis involves "nibbling" of portions of the donor cell membrane, resulting in intercellular membrane component transfer and potential donor cell death [149–151]. CD47 blockade enhances antitumor responses through trogocytosis in renal cell carcinoma and diffuse large B-cell lymphoma [152, 153]. CD24 represents an additional "do not eat me" protein overexpressed in certain malignancies. In high-grade serous ovarian carcinoma, tumor-associated mesothelin activates Wnt/β-catenin signaling, subsequently upregulating CD24 expression [154]. CD24/Siglec-10 signaling disrupts tumor cell immune surveillance, and pathway blockade promotes macrophage phagocytosis of CD24-expressing tumor cells, effectively inhibiting tumor growth. A novel intratumorally delivered dual inhibitor targeting both CD47/SIRPα and CD24/Siglec-10 pathways suppresses phagocytosis checkpoints on malignant cells, reactivates the TAM-CD8⁺ T cell axis, and reduces off-target risk [155].

#### Targeting metabolism

TAMs undergo extensive metabolic reprogramming within the TME, the reprogramming critically influences their pro-inflammatory or anti-inflammatory polarization, revealing important therapeutic opportunities [156, 157]. Recent studies show that obesity selectively upregulates PD-1 expression on TAMs [158]. Key obesity-associated inflammatory mediators induce macrophage PD-1 expression through mechanisms dependent on mTORC1 and glycolysis. PD-1 subsequently delivers negative feedback to TAMs, inhibiting glycolysis, phagocytosis, and T cell stimulatory capacity. PD-1 blockade elevates glycolytic activity in macrophages, which is essential for enhanced expression of CD86 and MHC-I/II molecules on TAMs and for boosting the T cell-activating function.

As the predominant cell type responsible for glucose uptake and metabolism in the TME, TAMs with enhanced glucose metabolism significantly contribute to tumor metabolite accumulation. In TNBC, targeted inhibition of the non-receptor tyrosine kinase HCK in TAMs modulates the PI3K/AKT-mTOR-HIF-1α pathway, shifting the metabolism from oxidative phosphorylation toward glycolysis [159]. The metabolic switch repolarizes TAMs to an anti-tumoral phenotype and sensitizes TNBC to immune checkpoint blockade. Lactate has emerged as a crucial TME regulator. In glioma, GPR65 is selectively highly expressed on TAMs, where it senses lactate via the cAMP/PKA/CREB pathway and stimulates HMGB1 secretion [160]. Interrupting the feedback loop represents a promising therapeutic strategy for GBM. Elevated lactate also drives transcriptional reprogramming in TAMs through the ENSA-STAT3 axis, reinforcing an immunosuppressive milieu [161]. Targeting ENSA-K63la augments ICB efficacy in pancreatic tumor models. Notably, succinate promotes classical anti-tumoral-like macrophage polarization by enhancing glycolysis and attenuating TCA cycle activity via protein succinylation, offering a distinct approach to modulate TAM polarization [162].

Tumor cells require substantial lipid resources for membrane synthesis, signaling, and energy storage. Cancer and high-cholesterol diets independently and synergistically activate RORγ-dependent expansion of myeloid-derived suppressor cells and pro-tumoral TAMs, facilitating cancer dissemination. Lowering cholesterol levels through genetic or pharmacological inhibition of PCSK9 suppresses MDSC expansion, pro-tumoral TAM accumulation, and tumor progression in a RORγ-dependent manner, thereby unleashing specific anti-tumor immunity [163]. In HCC, long-chain unsaturated fatty acids activate PPARγ via FABP5, conferring immunosuppressive properties on TAMs and promoting tumorigenesis. The discovery offers new insights into the immunomodulatory role of FABP5⁺ lipid-laden TAMs in HCC [164]. In vivo activation of NOD1 downregulates perilipin 5 expression, impairing fatty acid oxidation and inducing free fatty acid accumulation in TAMs [165]. The metabolic change promotes membrane localization of the costimulatory molecule OX40L in a lipid modification-dependent manner, leading to CD8⁺ T cell activation in HCC and improving anti-PD-1 therapy efficacy.

Amino acid metabolism in macrophages strongly influences pro-tumoral polarization. Polyamines derived from arginine metabolism enhance pro-tumor TAM polarization through thymine DNA glycosylase-mediated DNA demethylation, a process modulated by p53 signaling. Targeting the arginine-polyamine-TDG axis between cancer cells and macrophages significantly suppresses breast cancer growth, underscoring its therapeutic promise [166]. In HCC, spermidine primarily reprograms TAMs by activating the PI3K-Akt-mTOR-S6K pathway [167]. Combining spermidine inhibition with immune checkpoint blockade effectively reduces tumor burden in vivo*.*

To support rapid DNA and RNA synthesis, tumor cells markedly upregulate purine and pyrimidine production. Enhanced purine metabolism characterizes TAMs with pro-tumor and terminally differentiated phenotypes and correlates with poor response to immune checkpoint blockade [168]. CXCL8 secreted by non-small cell lung cancer (NSCLC) cells recruits macrophages from peritumoral tissues to tumor sites and stimulates purine metabolism by increasing xanthine dehydrogenase and uric acid production, thereby promoting PD-L1 expression. Moreover, purine metabolism-mediated macrophage immunosuppression depends on NLRP3/caspase-1/IL-1β signaling. Inhibiting purine metabolism enhances anti-tumor immunity and improves anti-PD-L1 therapy outcomes [169].

Collectively, the findings provide vital insights into metabolic crosstalk in tumors and highlight promising therapeutic strategies to overcome treatment resistance and improve patient prognosis. Additional research is warranted to explore the potential and effectiveness of metabolism-targeting approaches in cancer treatment.

#### CAR-M

Chimeric antigen receptor T-cell therapy has demonstrated limited efficacy against solid tumors due to poor tumor penetration, the immunosuppressive TME, and treatment-related adverse events [170]. CAR technology have been adapted for other innate immune cells. Macrophages represent an ideal platform due to their abundance and functional versatility within the TME [171].

CAR-M therapies primarily control tumor progression by enhancing phagocytosis, secreting cytokines essential for adaptive cytotoxic responses, and modifying the pro-metastatic TME [172]. Using CRISPR-Cas9 gene editing, an anti-GD2 CAR gene was integrated into mouse pluripotent stem cells, which were subsequently differentiated into macrophages designated anti-GD2 CAR-Ms [173]. The engineered cells phagocytosed GD2-expressing neuroblastoma and melanoma cells in vitro and suppressed neuroblastoma growth in mouse models. A lentiviral vector platform was developed to deliver interferon-alpha coding sequences to liver macrophages [174]. The genetically modified liver macrophages expressed IFN-α, activated T cells, and inhibited liver metastasis in mice. CT-0508, currently under evaluation in patients with relapsed/refractory HER2-overexpressing solid tumors, represents the first CAR-M cell therapy to enter clinical trials, marking a new era in macrophage-based therapeutics [175]. CAR-Ms developed from genetically modified human peritoneal macrophages specifically targeted HER2-expressing GC cells, triggered phagocytosis, significantly promoted tumor regression in HER2-positive models, extended overall survival (OS) in mouse models of peritoneal carcinomatosis, and offer a promising treatment option for HER2-positive GC patients [176]. CAR-Ms targeting tumor-specific antigens such as EGFRvIII, IL-13Rα2, and mesothelin have also demonstrated considerable potential for treating brain tumors [177]. CAR-M therapies directed against HER2 and EGFRvIII improved survival in preclinical models and are anticipated to cross the blood–brain barrier, while IL-13Rα2 and mesothelin have been validated as potential targets for brain tumor eradication. Genetically engineered macrophages carrying an oncolytic adenovirus were developed to maintain an anti-tumoral-like phenotype and deliver interleukin-12 and CXCL9 directly to local tumor sites, thereby reversing the immunosuppressive TME [178]. The approach provides a novel strategy for enhancing immunotherapy efficacy through macrophage-mediated gene delivery in TNBC.

Although CAR-M cells have yielded promising preclinical results, the clinical translation faces challenges including a scarcity of tumor-specific antigens, risks associated with gene transfer, and difficulties in achieving efficient in vivo delivery [179]. Ongoing research integrating nanotechnology, CRISPR, and other advanced technologies holds considerable promise for addressing the challenges in the foreseeable future [180, 181].

### Combination therapies

Combination therapies directed against TAMs constitute a precision oncology approach grounded in a sophisticated appreciation of TME complexity. While single-agent treatments often fail to counter the multifaceted tumor-promoting mechanisms orchestrated by TAMs, synergistic combinations of TAM-targeting agents with other therapeutic modalities enable multi-pathway attacks that collectively reverse immunosuppression and overcome treatment resistance.

The CSF1/CSF1R axis represents a promising therapeutic target for combination strategies. Cabiralizumab binds CSF1R and competitively inhibits ligand-receptor interaction [182]. However, cabiralizumab monotherapy demonstrates limited efficacy and presents adverse events, diminishing its appeal as a standalone treatment [183]. In a phase II trial, PC patients receiving cabiralizumab combined with nivolumab failed to meet the primary progression-free survival endpoint [184]. Similarly, the anti-CSF1 monoclonal antibody MCS110 combined with gemcitabine and carboplatin showed no benefit for TNBC patients [185]. A phase II trial evaluating MCS110 with BRAF/MEK inhibitors in melanoma patients is currently ongoing [186].

Systemic drug administration often causes off-target adverse effects, whereas nanotechnology facilitates precise drug delivery, enhances therapeutic efficacy, and minimizes off-target impacts. Integrating targeted nanoparticles with inhibitors or chemotherapy drugs selectively promote macrophage depletion or reprogramming, thereby maximizing anti-tumor outcomes. To generate the therapeutic cells, macrophages were polarized to an anti-tumoral phenotype using LPS and Ac4ManNAz and subsequently functionalized with liposomes containing a TLR7/8 agonist [187]. The engineered population, designated LAMΦ-M7/8a, demonstrated the ability to phagocytose 4T1 tumor cells and secrete high levels of IL-6 and TNF-α in vitro. When administered via either intratumoral or intravenous routes along with doxorubicin liposomes to 4T1-bearing mice, LAMΦ-M7/8a alleviated tumor burden. The treatment promoted a favorable immune remodeling in the TME, characterized by increased CD8^+^ T cell infiltration concurrently with a decrease in myeloid-derived suppressor cells [188]. A nanomodulator (Ft-E64/Hf@Lipo) combining the radiosensitizer hafnium with the cysteine protease inhibitor E64 synergistically revitalized TAM antigen presentation. The reprogrammed TAMs effectively presented tumor antigens and activated CD8⁺ T cells; this strategy combined with anti-PD-1 therapy demonstrated excellent efficacy against large, refractory CT26 tumors [189].

Combination immunotherapies represent another avenue for improving cancer treatment outcomes. The CD47 blocker TTI-621 is evaluated with anti-PD-1 antibody pembrolizumab in diffuse large B-cell lymphoma [186]. Bispecific antibody development shows considerable promise for cancer treatment. Unlike conventional monoclonal antibodies targeting single antigens, bispecific antibodies engage two different epitopes on the same cell, increase binding affinity, and modulate multiple signaling pathways [190]. The capability helps shape an anti-tumor TME, accompanied by altered immune cell infiltration and immune-related gene regulation [191]. The anti-CD47/anti-PD-L1 bispecific antibody IBI-322 has advanced to phase II trials in small cell lung cancer [192]. The third-generation CD47-SIRPα inhibitor HCB101 significantly inhibits tumor growth as monotherapy and demonstrates synergistic anti-tumor effects with anti-HER2 or anti-EGFR monoclonal antibodies [148]. Combined targeting of signal regulatory protein α with 4-1BB is also under investigation, highlighting the potential of bispecific antibodies in oncology for phenotype repolarization, phagocytosis promotion, and immunotherapy enhancement [153, 193–196]. SIRPα also constrains macrophage phagocytic function independently of CD47, suggesting additional molecular targets and signaling pathways [197].

TLRs are abundantly expressed in macrophages, and their activation offers a potential strategy for converting TAMs to an anti-tumoral-like phenotype [198]. TLR stimulation in TAMs coincides with increased PD-L1 expression that attenuates CD8⁺ T cell cytotoxicity, providing rationale for combining TLR agonists with PD-1 inhibitors. The TLR7 agonist LHC165 is being evaluated alone and with the PD-1 blocker spartalizumab in patients with advanced solid tumors [199].

Notch signaling upregulation in TAMs enhances the immunosuppressive activity [200–202]. In PC and TNBC, NOTCH1 inhibition combined with anti-PD-1 therapy reduces tumor growth and activates anti-tumor immunity. Given the dynamic role of interleukins and the receptors in macrophage polarization and TME remodeling, modulating the pathways by enhancing pro-inflammatory signals or inhibiting immunosuppressive mechanisms represents a promising strategy. Targeting the IL-33/ST2 axis reprograms the TME and enhances anti-PD-L1 responses in lung cancer and melanoma [203, 204]. The probiotic Clostridium butyricum expresses surface protein SecD, which binds the CRC cell receptor GRP78, inactivating the PI3K-AKT-NF-κB pathway. This reduces interleukin-6 secretion, thereby activating cytotoxic CD8⁺ T lymphocytes and impairing TAMs to enhance anti-PD-1 efficacy in CRC [205]. In HCC, the non-N-terminal fragment of GSDME in macrophages binds PDPK1, activating the PI3K-AKT pathway and promoting pro-tumoral-like polarization [206]. The small molecule eliprodil inhibits GSDME-mediated PDPK1 phosphorylation, and its combination with anti-PD1 provides a promising immunotherapeutic strategy. TAMs activate NF-κB through TNF-α/IL-1β secretion in the liver microenvironment, transcriptionally upregulating OTU deubiquitinase 1 expression, which stabilizes FGL1 via deubiquitination. Disrupting the TAM-OTUD1-FGL1 axis suppresses metastatic CRC progression and acts synergistically with immune checkpoint blockade [207].

TAM-targeting strategies combined with chemotherapy, radiotherapy, and ICIs offer promising therapeutic opportunities. Future advances will increasingly rely on precise biomarker identification, optimized treatment scheduling and sequencing, and next-generation drugs specifically targeting intratumoral TAM subpopulations. TAM-centered combination therapy represents the next frontier in cancer immunotherapy, providing a transformative paradigm for achieving more effective and durable tumor control through systematic remodeling of the TME.

### Advances in drug development

CSF1R represents a promising target in contemporary drug development. Multiple strategies have been employed to modulate CSF1R signaling, such as small molecules that inhibit its tyrosine kinase activity and antibodies that bind CSF1R to block ligand-receptor interaction or disrupt receptor dimerization [208, 209] (Table 1). Pexidartinib, a receptor tyrosine kinase inhibitor targeting CSF1R [135], exhibited favorable tolerability, pharmacokinetics, and pharmacodynamics in the first-in-human Phase I trial for solid tumors [210]. Phase I study evaluated the safety and efficacy of pexidartinib combined with durvalumab (anti-PD-L1) in patients with advanced colorectal and PC, with the aim of enhancing response to PD-L1 blockade by depleting CSF-1-dependent inhibitory TAMs [211]. Results revealed that pexidartinib impaired DC differentiation via FLT3 signaling inhibition, which explains the limited clinical antitumor activity observed. The findings suggest that FLT3 inhibition should be taken into account when combining TKIs with ICIs. Pexidartinib has been utilized in the treatment of several cancer types, and clinical trials continue [212–214, 224]. Following a completed Phase III trial [215, 225], the U.S. FDA approved pexidartinib for tenosynovial giant cell tumor (TGCT). HMPL-012 (sulfatinib), a novel TKI, received approval in China in 2020 for extra-pancreatic neuroendocrine tumors based on completed Phase III trials and is now under evaluation in clinical trials for various cancers [135, 216–218, 226]. DCC-3014, another potent CSF1R inhibitor, is also in clinical development [219–221, 227]. RG7155 remains active in Phase III trials and has been evaluated for TGCT [222]. A novel potent CSF1R inhibitor, PXB17, significantly blocked PI3K/AKT/mTORC1 signaling activation, reprogrammed pro-tumoral macrophages toward an anti-tumoral phenotype, induced CD8^+^ T cell infiltration into tumors, and ameliorated the immunosuppressive microenvironment [228]. In vivo, PXB17 inhibited CRC growth more effectively than PLX3397. Efforts to target the CSF1R ligand CSF1 have been limited. MCS110, a humanized monoclonal antibody that binds CSF1 and blocks downstream signaling [229], was administered as monotherapy in TGCT and reduced tumor size [223]. However, patient numbers were small, and further studies are required to confirm its efficacy in TGCT.

**Table 1:** Clinical trials targeting CSF1/CSF1R for TAM treatment

TAM target	NCT number	Study title	Interventions	Phases		Refs
CSF1R	NCT02471716	Study of Cabiralizumab in Patients With Pigmented Villonodular Synovitis/Diffuse Type Tenosynovial Giant Cell Tumor (FPA008-002)	Biological: FPA008	Phase 1/2		[183]
CSF1R	NCT03336216	A Study of Cabiralizumab Given With Nivolumab With and Without Chemotherapy in Patients With Advanced Pancreatic Cancer	Biological: Cabiralizumab Drug: Nab-paclitaxel Drug: Onivyde Biological: Nivolumab Drug: Fluorouracil Drug: Gemcitabine Drug: Oxaliplatin Drug: Leucovorin Drug: Irinotecan Hydrochloride	Phase 2		[184]
CSF1	NCT02435680	Efficacy Study of MCS110 Given With Carboplatin and Gemcitabine in Advanced Triple Negative Breast Cancer (TNBC)	Drug: MCS110 Drug: carboplatin Drug: gemcitabine	Phase 2		[185]
CSF1	NCT03455764	MCS110 With BRAF/MEK Inhibition in Patients With Melanoma	Drug: MCS110 Drug: Dabrafenib Drug: Trametinib	Phase 1/2		[186]
CSF1R	NCT01004861	Safety Study of PLX108-01 in Patients With Solid Tumors	Drug: PLX3397	Phase 1		[210]
CSF1R	NCT02777710	Evaluation of Safety and Activity of an Anti-PDL1 Antibody (DURVALUMAB) Combined With CSF-1R TKI (PEXIDARTINIB) in Patients With Metastatic/Advanced Pancreatic or Colorectal Cancers (MEDIPLEX)	Drug: Pexidartinib Drug: Durvalumab	Phase 1		[211]
CSF1R	NCT04488822	A Study of the Efficacy and Safety of Pexidartinib in Adult Subjects With TGCT (PLX3397)	Drug: Pexidartinib	Phase 3		[212]
CSF1R	NCT01349036	A Phase 2 Study of PLX3397 in Patients With Recurrent Glioblastoma	Drug: PLX3397	Phase 2		[213]
CSF1R	NCT04703322	A Study of Pexidartinib in Tenosynovial Giant Cell Tumor in Japan	Drug: Pexidartinib		Phase 2	[214]
CSF1R	NCT02371369	Phase 3 Study of Pexidartinib for Pigmented Villonodular Synovitis (PVNS) or Giant Cell Tumor of the Tendon Sheath (GCT-TS) (ENLIVEN)	Drug: Pexidartinib Drug: Placebo		Phase 3	[215]
CSF1R	NCT02589821	Phase III Study of Surufatinib in Treating Advanced Pancreatic Neuroendocrine Tumors	Drug: Surufatinib Other: Placebo		Phase 3	[216]
CSF1R	NCT06329947	A Phase II Study of Surufatinib Combined With Camrelizumab and mFOLFOX6 as Second-line Treatment for Advanced PRAD	Drug: Surufatinib 250 mg/d qd once daily		Phase 2	[217]
CSF1R	NCT05236699	A Clinical Study of DEB-TACE Combined With Surufatinib and Camrelizumab in the Treatment of Inoperable or Metastatic ICC (CCGLC-005)	Combination Product: DEB-TACE combined with Surufatinib and Camrelizumab		Phase 2	[218]
CSF1R	NCT05059262	Study of Vimseltinib for Tenosynovial Giant Cell Tumor (MOTION)	Drug: Vimseltinib Drug: Placebo		Phase 3	[219]
CSF1R	NCT05723055	Evaluating Combination of Nivolumab and Axatilimab in Patients With Relapsed/Refractory Classical Hodgkin Lymphoma (NAHL)	Drug: Axatilimab Drug: Nivolumab		Phase 2	[220]
CSF1R	NCT05491226	Reinvigorating TNBC Response to Immunotherapy With Combination Myeloid Inhibition and Radiation	Drug: Pembrolizumab Radiation: Radiation Therapy Drug: Axatilimab		Phase 2	[221]
CSF1R	NCT05417789	Study of Emactuzumab for TGCT (TANGENT)	Drug: Emactuzumab Drug: Placebo		Phase 3	[222]
CSF1	NCT01643850	MCS110 in Patients With Pigmented Villonodular Synovitis (PVNS)	Drug: MCS110 Drug: Placebo		Phase 2	[223]

*Abbreviations*: *TAM* tumor-associated macrophage, *NCT* National Clinical Trial, *TGCT* tenosynovial giant cell tumor, *TANGENT* tumor alteration in normal and germline estimation tool, *PRAD* prostate adenocarcinoma, *ICC* intrahepatic cholangiocarcinoma

Disrupting chemokine receptor axes is considered a viable strategy to improve cancer therapy [230]. Several monoclonal antibodies and receptor antagonists targeting monocyte recruitment have been developed (Table 2). Despite multiple clinical trials, most chemokine-targeted agents remain stalled in Phase II and have not been adopted clinically, either as monotherapy or in combination. Carlumab, one of the earliest antibodies studied, binds and neutralizes CCL2. In a Phase II trial in prostate cancer, no prostate-specific antigen or RECIST responses were observed [238]. Carlumab reduced serum CCL2 levels within 24 h, but concentrations rebounded to baseline or higher by day 8, likely due to compensatory chemokine release that has contributed to treatment failure [231]. Plozalizumab, an anti-CCR2 monoclonal antibody that blocks the CCL2-CCR2 axis, was discontinued after its clinical trial was terminated early owing to severe adverse events [232, 233]. The small-molecule CCR2/CCR5 antagonist BMS-813160 is currently in Phase II studies for NSCLC and HCC [234]. The CXCL12-CXCR4 axis represents another target for inhibiting leukocyte recruitment. Olaptesed Pegol, a pegylated RNA oligonucleotide, binds CXCL12 with high affinity and blocks its interaction with CXCR4. Its safety and pharmacokinetics have been assessed, and it is anticipated to enter Phase II studies in metastatic PC [235]. Several CXCR4 antagonists have also been developed. BL-8040 completed Phase II evaluation in PC [236], and AMD3100 has been assessed in Phase II trials for GBM [237].

**Table 2:** Clinical trials targeting chemokine receptor axes for TAM treatment

TAM target	NCT number	Study title	Interventions	Phases	Refs
CCL2	NCT01204996	A Study of the Safety and Efficacy of CNTO 888 in Combination With SoC (Standard of Care) Chemotherapy in Patients With Solid Tumors	Drug: CNTO888 + DOXIL®/Caelyx® doxorubicin HCl liposome injection Drug: CNTO888 + gemcitabine Drug: CNTO888 + Paclitaxel and carboplatin Drug: CNTO888 + docetaxel	Phase 1	[231]
CCR2	NCT01015560	S0916, MLN1202 in Treating Patients With Bone Metastases	Drug: anti-CCR2 monoclonal antibody MLN1202 Genetic: polymorphism analysis Other: laboratory biomarker analysis	Phase 2	[232]
CCR2	NCT02723006	Study to Evaluate the Safety, Tolerability, and Pharmacodynamics of Investigational Treatments in Combination With Standard of Care Immune Checkpoint Inhibitors in Participants With Advanced Melanoma	Drug: TAK-580 Drug: TAK-202 Drug: vedolizumab Drug: nivolumab Drug: ipilimumab	Phase 1	[233]
CCR2/5	NCT04123379	Neoadjuvant Nivolumab with CCR2/5-inhibitor or Anti-IL-8) for Non-small Cell Lung Cancer (NSCLC) or Hepatocellular Carcinoma (HCC)	Drug: Nivolumab Drug: BMS-813160 Drug: BMS-986253	Phase 2	[234]
CXCL12	NCT04901741	Olaptesed With Pembrolizumab and Nanoliposomal Irinotecan or Gemcitabine/​Nab-Paclitaxel in MSS Pancreatic Cancer (OPTIMUS)	Drug: Olaptesed pegol Drug: Pembrolizumab	Phase 2	[235]
CXCR4	NCT04543071	Chemo4METPANC Combination Chemokine Inhibitor, Immunotherapy, and Chemotherapy in Pancreatic Adenocarcinoma	Drug: Motixafortide Drug: Cemiplimab Drug: Gemcitabine Drug: Nab paclitaxel	Phase 2	[236]
CXCR4	NCT03746080	Whole Brain Radiation Therapy With Standard Temozolomide Chemo-Radiotherapy and Plerixafor in Treating Patients With Glioblastoma	Drug: Plerixafor Drug: Temozolomide Radiation: Whole-Brain Radiotherapy (WBRT) Radiation: Radiation Therapy	Phase 2	[237]

*Abbreviations: TAM* tumor-associated macrophage, *NCT* National Clinical Trial

Multiple antibodies targeting CD47 have been developed to prevent SIRPα activation and reprogram TAMs for tumor cell phagocytosis [239, 240] (Table 3). Hu5F9-G4 was a first-in-class anti-CD47 antibody evaluated in Phase II trials for several solid tumors [241–243]. However, in 2024, the FDA placed a clinical hold on magrolimab studies in acute myeloid leukemia and myelodysplastic syndromes due to lack of efficacy and an increased risk of death, potentially halting its Phase III development [244]. Lemzoparlimab, another anti-CD47 antibody, is in Phase III studies for myelodysplastic syndromes [245]. SIRPα-Fc fusion proteins, which consist of a modified SIRPα domain fused to the Fc region of human IgG, retain CD47-binding capacity and abrogate CD47/SIRPα-mediated inhibition of phagocytosis [251]. A Phase II/III trial of the SIRPα-Fc fusion protein evorpacept (ALX-148) has been initiated [246–250].

**Table 3:** Clinical trials targeting CD47 for TAM treatment

TAM target	NCT number	Study title	Interventions	Phases	Refs
CD47	NCT04854499	Study of Magrolimab Combination Therapy in Patients With Head and Neck Squamous Cell Carcinoma (ELEVATE HNSCC)	Drug: Magrolimab Drug: Pembrolizumab Drug: Docetaxel Drug: 5-FU Drug: Cisplatin Drug: Carboplatin Drug: Zimberelimab	Phase 2	[241]
CD47	NCT05330429	Study of Magrolimab Given Together With FOLFIRI/Bevacizumab (BEV) in Participants With Previously Treated Advanced Inoperable Metastatic Colorectal Cancer (mCRC) (ELEVATE CRC)	Drug: Magrolimab Drug: Bevacizumab Drug: Irinotecan Drug: Fluorouracil Drug: Leucovorin	Phase 2	[242]
CD47	NCT04958785	Study of Magrolimab Combination Therapy in Patients With Non-Surgically Removable Locally Advanced or Metastatic Triple-Negative Breast Cancer (ELEVATE TNBC)	Drug: Magrolimab Drug: Nab-Paclitaxel Drug: Paclitaxel Drug: Sacituzumab Govitecan-hziy	Phase 2	[243]
CD47	NCT05079230	Study of Magrolimab Versus Placebo in Combination With Venetoclax and Azacitidine in Participants With Acute Myeloid Leukemia (ENHANCE-3)	Drug: Magrolimab Drug: Venetoclax Drug: Azacitidine Drug: Placebo	Phase 3	[244]
CD47	NCT05709093	A Phase 3 Clinical Study to Evaluate Lemzoparlimab for Injection in Combination With Azacitidine (AZA) Versus AZA Monotherapy in Treatment-naïve Patients With Higher-risk Myelodysplastic Syndrome (MDS)	Drug:Lemzoparlimab + Azacitidine (AZA) Drug:Azacitidine (AZA)	Phase 3	[245]
CD47	NCT04675294	Evorpacept (ALX148) in Combination With Pembrolizumab in Patients With Advanced Head and Neck Squamous Cell Carcinoma (ASPEN-03)	Drug: evorpacept Drug: pembrolizumab	Phase 2	[246]
CD47	NCT05787639	Neoadjuvant Immunoradiotherapy With Evorpacept and Pembrolizumab in HPVOPC (Human Papilloma Virus Oropharynx Cancer)	Drug: Evorpacept	Phase 2	[247]
CD47	NCT05467670	Safety and Efficacy of Anti-CD47, ALX148 in Combination with Liposomal Doxorubicin and Pembrolizumab in Recurrent Platinum-resistant Ovarian Cancer	Drug: Pembrolizumab Drug: ALX148 Drug: Doxorubicin	Phase 2	[248]
CD47	NCT05167409	A Study of Evorpacept (ALX148) With Cetuximab and Pembrolizumab for Refractory Microsatellite Stable Metastatic Colorectal Cancer	Drug: Evorpacept (ALX148) Drug: Cetuximab Drug: Pembrolizumab	Phase 2	[249]
CD47	NCT05002127	A Study of Evorpacept (ALX148) in Patients With Advanced HER2 + Gastric Cancer (ASPEN-06)	Drug: Evorpacept (ALX148) Drug: Trastuzumab Drug: Ramucirumab Drug: Paclitaxel	Phase 2/3	[250]

*Abbreviations: TAM* tumor-associated macrophage, *NCT* National Clinical Trial, *HER2* + human epidermal growth factor receptor 2 positive

TLR agonists targeting TLR3, TLR4, TLR7/8, and TLR9 have been investigated as cancer therapeutics in clinical trials. The TLR3 agonist rintatolimod is being administered intravenously in a currently recruiting Phase II trial for PC [252]. BO-112, another TLR3 agonist, is delivered intratumorally in combination with intravenous pembrolizumab in melanoma patients [253]. In preclinical studies, protein canopy homolog 2 (CNPY2) was identified as a novel TLR4 modulator that promotes macrophage cytokine production. CNPY2 binds directly to TLR4; deficiency in CNPY2 reduces TLR4 cell surface expression, inhibits NF-κB2/p52-mediated transcription of Flt1 and Kdr, diminishes cytokine production, and suppresses hepatocarcinogenesis [254]. The TLR7 agonist TMX-101 is administered intravesically for bladder cancer. A Phase II study demonstrated negative cytology and biopsy results in 20% of patients after 6 weeks, although larger cohorts are needed for validation [255]. The TLR8 agonist motolimod was administered subcutaneously in Phase II studies [256]; addition of motolimod to pegylated liposomal doxorubicin did not improve overall or progression-free survival [257]. Intratumoral injection of the TLR9 agonist CMP-001 in melanoma patients resulted in partial responses in 46.7% of patients [258], and a Phase II/III study in melanoma is ongoing [259].

Chronic activation of inflammatory pathways is a hallmark of immunosuppressive tumors. STAT3, a key transcription factor downstream of JAK signaling in macrophage polarization, has been targeted by small-molecule inhibitors employing diverse mechanisms [260]. OPB-31121 inhibits STAT3 phosphorylation and subsequent target promoter activation. It was well tolerated in a Phase I/II study in HCC patients, but no further development information is available [261]. WP1066, another STAT3 inhibitor, blocks nuclear translocation of phosphorylated STAT3 and inhibits STAT3-mediated transcriptional activation; it is currently being evaluated in a Phase II trial for GBM in combination with radiotherapy [262]. Eganelisib, a potent and selective small-molecule PI3K-γ inhibitor, preclinically reshapes the TME by reducing myeloid cell recruitment and reprogramming TAMs from an immunosuppressive to an immunostimulatory phenotype, thereby enhancing ICI activity. A Phase II study combining eganelisib with atezolizumab and nab-paclitaxel as first-line treatment for metastatic TNBC yielded results that support its role as a TAM-reprogramming immunotherapy and provide a rationale for its combination with ICIs and chemotherapy in TAM-driven, ICI-resistant indications [263]. Bintrafusp alfa, a first-in-class bifunctional fusion protein targeting TGF-β and PD-L1, showed encouraging efficacy as a second-line therapy for NSCLC patients in dose-expansion cohorts of an open-label Phase I trial. It demonstrated modest clinical activity and a manageable safety profile, accompanied by significant immune changes consistent with TME modulation [264].

Systemic targeting of TAMs using nanomedicines represents a promising cancer therapy approach due to its high specificity and reduced side effects. TAMs exhibit a natural propensity for nanoparticle uptake, internalizing them at rates up to ten times greater than tumor cells [109]. Delivering diverse agents to TAMs via nanocarriers further reshapes the immune microenvironment and enhance antitumor immunity. Co-delivery of an IL-12-expressing gene and the CSF1R inhibitor PLX3397 using nanotechnology effectively activated T cell-mediated immunity and reduced pro-tumoral TAM numbers more significantly than delivery of either agent alone [265]. The CD47-specific anticancer polypeptide RS17 was hybridized with anti-tumoral macrophage-derived nanovesicles (Spi@hel-RS17) to effectively inhibit CD47-SIRPα signaling [266]. A fluorinated proteolysis-targeting chimera-sorafenib nano-assembly (FCP@SF/FPro) was developed. The innovative system incorporates a fluorinated proteolysis-targeting chimera targeting BRD4 (FPro) and sorafenib within a fluorinated PEG-conjugated polyethylenimine (FCP) matrix, stabilized by fluorine, ensuring high drug loading and stability [267]. By rapidly degrading BRD4, FCP@SF/FPro induced apoptosis, downregulated HIF-1α to attenuate hypoxia signaling, alleviated immunosuppression by reducing PD-L1 expression, and increased the anti-tumoral/pro-tumoral TAM ratio, underscoring its innovative potential as a combination therapy for resistant HCC.

The development of therapeutics targeting TAMs has evolved into a multidimensional endeavor addressing the recruitment, survival, polarization, and function, marking the advent of a new era centered on combination therapies and precision targeting. From traditional cell depletion to functional reprogramming, and further to innovative modalities such as cell therapy and targeted delivery, the strategies are systematically reshaping the TME, offering promising directions for overcoming tumor immune resistance and treatment refractoriness.

## Applications of TAMs in diagnosis and prognosis

During tumor progression, TAMs display spatiotemporal plasticity, dynamically transitioning between tumor-suppressive and tumor-promoting functions. The dual functional nature contributes to subtype-specific clinical presentations and treatment outcomes, underscoring the potential of TAMs as valuable biomarkers and the critical influence on patient prognosis.

### TAMs as biomarkers

As pivotal immune components, TAMs engage with diverse elements within the TME [268]. Accumulating evidence on the interplay between TAMs and tumors has uncovered distinctive TAM traits during carcinogenesis. When TAMs interact with or alter the tumor ECM, they frequently adopt an pro-tumoral-polarized phenotype [269], pointing to the relevance in early tumor detection, treatment strategies, and prognostic evaluation.

Among the most established biomarkers are members of the scavenger receptor family, such as CD206, CD163, stabilin-1, Marco, CD36, and CD204 [270, 271], which are expressed on CD68^+^ macrophages in tumor tissues [272–274]. CD206 and CD163 represent the most frequently used markers for pro-tumoral-polarized TAMs and are linked to metastatic behavior and unfavorable outcomes across multiple cancers [275, 276]. CD163 expression also shows a positive correlation with the degree of serum or lymphatic metastasis [276], supporting its utility as a promising biomarker. Single-cell transcriptomic analysis of samples revealed a prominent pro-tumoral-like phenotype in TAMs, reflecting the immunosuppressive role in the TME and suggesting the potential as predictors of GC [277]. CD36, a recently characterized scavenger receptor in TAMs, participates in immunosuppression. Through p38 activation downstream of oxidized lipid signaling, CD36 serves as an endogenous inhibitor of type I interferon signaling in macrophages, revealing a potential therapeutic strategy to promote interferon production and improve immunotherapy responses [278].

Emerging functional biomarkers including TREM2, SPP1, SPI1, C1Q, S1P, CXCL9, NUPR1, MS4A4A, Siglec-G/10 and BST2, have been proposed as indicators of immunosuppression, lipid metabolism, phagocytosis, antigen presentation, glycolysis, angiogenesis, hypoxia, and tumor cell invasion in TAMs across various malignancies [85, 279–289]. TREM2, a surface lipid receptor, has arisen as a novel and potent macrophage biomarker with marked immunosuppressive activity [290, 291]. GPNMB-positive TAMs serve as a specific hallmark of GBM and correlate with poor patient survival [292, 293]. SPP1hi TAMs act as central mediators of ICI resistance in metastatic castration-resistant prostate cancer by engaging adenosine signaling, highlighting the dual relevance as therapeutic targets and predictive biomarkers [294]. HO-1-expressing TAMs mark invasive tumor regions and have been localized to the advancing edges in both human melanomas and murine models [295]. Zeb2, a principal regulator of TAM programming, orchestrates immunosuppression by dampening type I interferon signaling and antigen presentation while reinforcing inhibitory pathways [296]. Elevated Zeb2 expression in macrophage-dense human tumors is correlated with adverse outcomes. In LUAD, LTβR sustains TAM immunosuppression and pro-tumoral traits via non-canonical NF-κB and Wnt/β-catenin signaling [297]. Infiltration by LTBR^+^ TAMs is linked to advanced disease stage, ICI non-response, and inferior survival in LUAD. Recently, CXCL9 and SSP1 were identified as biomarkers specific to discrete TAM subsets in HNSCC [298]. The transcriptional signatures of CXCL9^+^ and SSP1^+^ TAMs do not conform to the conventional anti-tumoral/pro-tumoral dichotomy. The observation reinforces that the classical anti-tumoral/pro-tumoral framework is overly simplistic and emphasizes the need for a refined appreciation of TAM heterogeneity to advance targeted therapeutic approaches.

### Correlation between TAMs and patient prognosis

TAMs constitute the most prevalent immune cell population in the tumor immune microenvironment. The level of infiltration is strongly linked to tumor stage and metastatic potential. In the majority of solid tumors, high TAM density is associated with an unfavorable prognosis [299]. For example, breast cancer patients with higher pathological grades show increased TAM levels and experience significantly shorter OS and disease-free survival compared to patients with lower TAM infiltration [300]. Specifically, the presence of CD163⁺ and CD204⁺ TAM subsets is correlated with poor clinical outcomes, as the populations are implicated in rapid tumor proliferation and poor differentiation [301]. In melanoma, elevated TAM numbers are associated with increased tumor recurrence rates [302]. The effect is partly attributed to the ability of TAMs to suppress CD8⁺ T cell activity, thereby diminishing tumor clearance. However, the association between TAM infiltration and prognosis remains inconsistent in CRC. Some studies have demonstrated that CD163 is differentially expressed in CRC tissues and serves as a marker of poor prognosis [303]. A high CD163⁺/CD68⁺ ratio or an elevated pro-tumoral/anti-tumoral ratio predicts lymphatic metastasis in CRC patients [304, 305]. Additionally, high levels of C1Q⁺ TPP1⁺ TAMs in CRC tissues correlate with reduced survival probability [283]. Conversely, other research findings indicate a positive correlation between TAM density and favorable prognosis in CRC [306, 307]. CD68^+^ TAM numbers are decreased in patients with advanced-stage CRC, regional lymph node metastasis, or distant metastasis, and the reduction is linked to improved survival rates. The discrepant findings underscore the ongoing debate regarding the prognostic value of TAMs in CRC, which is influenced by underlying tumor heterogeneity.

The spatial distribution and density of TAMs significantly impact patient prognosis [308]. In lung cancer studies, while the overall density of anti-tumoral TAMs was generally higher than that of pro-tumoral TAMs, pro-tumoral TAMs were found to be more concentrated at the invasive margin [309]. No statistically significant difference in anti-tumoral TAM density was observed between the tumor center and the invasive margin. The risk of metastasis increased significantly with greater distance from either pro-tumoral tumor center-TAMs or pro-tumoral invasive margin-TAMs, and tumor size showed a correlation with proximity to pro-tumoral invasive margin-TAMs, with larger tumors tending to be situated closer to the cells. In GC, TAM distribution varies across different immune microenvironments, influencing tumor initiation and progression and, consequently, patient outcomes [310]. Macrophage counts have been correlated with both recurrence-free survival and OS. Therefore, targeting the spatial localization and abundance of TAMs within the TME represents a promising new direction for TAM-focused therapies.

Emerging biomarkers continue to clarify the relationship between TAMs and prognosis, with high expression of these markers often predicting poor outcomes [25, 117, 311–313]. In solid tumors, the abundance of TREM2⁺ TAMs is associated with reduced survival or poor response to PD-1-based ICIs [26, 291, 314]. In prostate cancer, apolipoprotein E (APOE) in the TME binds to TREM2 on macrophages and promotes androgen receptor (AR) expression, which further upregulates the transcription of immune mediators such as IL-10, TGF-β1, IL-23A, and CCL2. As a result, AR, TREM2, and APOE are overexpressed in prostate cancer and correlate with poor prognosis [315] The role of SPP1-expressing TAMs in tumor progression is under active investigation, and high SPP1 expression is linked to an unfavorable prognosis [55, 316, 317]. In HNSCC, the ratio of CXCL9⁺ to SSP1⁺ TAMs, termed the CS score, shows a positive correlation with infiltration by T cells, B cells, and DCs [298]. Similarly, IL-8 has emerged as a promising biomarker and a potential therapeutic target for improving treatment efficacy [318, 319]. Clinically, the ChREBP-SP1-choline metabolism axis is associated with adverse clinical outcomes in CRC [319]. In clear cell renal cell carcinoma, the transcription factor CEBPD, which regulates TAM polarization, is linked to a decreased anti-tumoral/pro-tumoral ratio and worse clinical outcomes. CLEVER-1, predominantly expressed on TAMs in GC, is correlated with poor clinical outcomes, and high CLEVER-1⁺ TAM infiltration is associated with poor or adaptive resistance to PD-1 blockade [320]. Enriched C1q⁺ TAMs and elevated C1q expression are both associated with disease progression and poor prognosis in nasopharyngeal carcinoma [321]. Breast cancer patients exhibiting an ACVRL1ʰⁱ TAM signature have significantly shorter survival, and ACVRL1 has been identified as an effector target for adjuvant anti-angiogenic immunotherapy in metastatic models [322]. The transcription factor EHF recruits and activates TAMs via the CCL2/CCR2 axis, remodeling the TME, and serves as a potential prognostic biomarker in cholangiocarcinoma [323]. Notably, higher expression of SHISA3 in anti-tumor TAMs is correlated with better OS in lung cancer patients [324]. Additionally, SP140 is highly expressed in TAMs across multiple cancer types, including HNSCC. Interestingly, elevated SP140 expression correlates with higher tumor mutation burden, improved survival, and more favorable responses to immunotherapy [325].

Immunotherapy has become a standard approach in cancer treatment [326]. Although ICIs have demonstrated success in numerous clinical trials, only a limited proportion of patients exhibit a favorable response. The lack of ICI efficacy arise from inhibitory interactions between TAMs and T cells. In NSCLC, low intratumoral infiltration of CD163⁺ TAMs during anti-PD-1/PD-L1 therapy is associated with prolonged progression-free survival (PFS) and OS [327]. The TREM2⁺ TAM subset is correlated with poor prognosis and reduced response to PD-1-targeted therapy [328]. EGFR-TKIs demonstrate clinical efficacy in NSCLC. The third-generation TKI osimertinib exerts off-target immunomodulatory effects by activating the NLRP3 inflammasome in macrophages, thereby linking mitochondrial-lysosomal crosstalk to antitumor immunity. NLRP3 and IL-1β in TAMs serve as predictive biomarkers for EGFR-TKI efficacy, and combination therapy with recombinant IL-1β represents a novel strategy to enhance clinical outcomes [329]. In the ESCC microenvironment under immunotherapy, a feedback loop involving IFN-γ, CCL5ʰⁱ macrophages, and CD8⁺ T cells has been identified. Biomarkers associated with CCL5ʰⁱ macrophages correlate with better responses to immunotherapy in ESCC patients.

A consensus on the role of TAMs in anticancer treatment response and prognosis has not yet been established, as it varies depending on patient cohort, cancer type, and treatment regimen. The identification of TAM-based predictive biomarkers and accurate prognostic assessment are complicated by clinical challenges in tumor characterization before and after therapy, which accounts for discrepancies across studies. Nonetheless, deciphering TAM heterogeneity and elucidating TAM-mediated antitumor immunity have become urgent priorities in immunotherapy. A thorough understanding of the molecular events and metabolic reprogramming associated with TAMs in the TME will greatly advance our knowledge of treatment resistance and contribute to improved cancer therapeutics.

## Conclusion and prospects

Tumor pathogenesis unfolds as a complex, multi-step process influenced by numerous factors. TAMs constitute a major cellular component of the TME and account for a significant proportion of immune cells within it. Through intrinsic regulatory programs and dynamic crosstalk with stromal and other immune cells, TAMs actively contribute to the establishment of an immunosuppressive microenvironment, thereby participating in the full spectrum of tumor development, from initiation and progression to metastasis. Given the pivotal role in tumor progression, TAM-directed approaches for tumor prediction, prognostic evaluation, and targeted therapy have gained increasing attention. Thus, elucidating the precise regulatory mechanisms governing TAMs and identifying macrophage-specific targets are essential for refining current immunotherapeutic strategies.

Recent research has progressively unveiled the regulatory functions of TAMs in tumor progression and treatment response, spurring the exploration of TAM-targeting therapies. Several strategic approaches have been investigated, including limiting TAM recruitment into the TME, depleting existing TAM populations, and reprogramming their functional states. Although preclinical and clinical advances have validated TAMs as a viable therapeutic target, considerable challenges persist. The stem from the high degree of heterogeneity and plasticity exhibited by TAMs across tumor types, disease stages, and anatomical sites; the diversity among tumor subtypes and clinical staging complexity; along with hurdles in personalizing treatments and the substantial costs involved in developing engineered macrophage therapies. Many promising strategies remain in exploratory or early-phase clinical trials, constrained by the reliance on animal models and limited translatability to human TAM biology and patient-specific immune contexts. Refining preclinical models, using patient-derived organoids or humanized mouse systems improves translational relevance. The inherent complexity of TAMs, including the diverse cellular origins, post-differentiation heterogeneity, and context-dependent functional states throughout disease progression, complicates the identification of optimal therapeutic windows and precise molecular targets. While current clinical efforts remain largely centered on PD-L1 checkpoint blockade, this underscores the pressing need for combination strategies that incorporate novel immune checkpoint targets with conventional treatment modalities.

A central challenge in targeted therapy involves preventing off-target effects, as healthy macrophages residing across various tissues may express molecules also present on TAMs. Thus, achieving selective targeting of TAMs while sparing tissue-resident macrophages is essential to reduce adverse events and ensure both safety and treatment efficacy. An expanding repertoire of cancer-specific biomarkers offers a path toward the precision needed to direct therapeutics to relevant cellular subsets. However, strategies such as targeting inflammatory cytokines, blocking inhibitory receptors, enhancing antigen presentation, modulating metabolic pathways, or reversing T-cell exhaustion with ICIs carry the risk of immune-related adverse events, wherein immune cells mistakenly attack healthy tissues. Emerging modalities like CAR-M cells mark a paradigm shift, genetically engineering macrophages to express chimeric antigen receptors has enabled the creation of cells that not only selectively target tumors but also contribute to reprogramming the TME. In parallel, nanobiotechnology presents transformative opportunities to overcome the limitations by enabling targeted drug delivery, spatiotemporal control over therapeutic release, and synergistic immunomodulation.

Categorizing TAMs into a binary anti-tumoral/pro-tumoral framework represents an oversimplification. Phenotypic gradations and transitional states, influenced by multiple factors that differ across cancer types and individual patients, add complexity to devising effective therapeutic strategies. Advances in single-cell technologies have uncovered numerous TAM subpopulations, each defined by distinct transcriptional signatures and clinical implications. The discoveries contest conventional classification systems, revealing context-dependent subsets that resist straightforward categorization and highlighting the need for a more refined appreciation of TAM heterogeneity. Deeper insights into TAM subtypes and the associated biomarkers will help decipher this diversity and assist in overcoming existing barriers in targeted therapy. Single-cell omics has further illuminated precise molecular and metabolic crosstalk within the TME, facilitating the identification of pivotal mechanisms behind TAM-modulated anti-tumor immunity and uncovering novel targets to enhance the clinical utility of targeted treatments.

Future studies should leverage multi-omics approaches, integrating single-cell analyses with spatial transcriptomics to fully resolve TAM heterogeneity. Elucidating the detailed molecular events and metabolic adaptations orchestrated by TAMs within the TME that govern tumor progression and therapy response will be instrumental in developing more effective, individualized, and precise combination treatments. To mitigate risks associated with targeted strategies and maximize therapeutic benefit, integrating TAM-focused interventions with safer, multimodal immunotherapies tailored to the tumor-specific immune context will be key to advancing oncology care. The construction of predictive models based on relevant genetic signatures represents a promising avenue for research, guiding the design of stratified and personalized combination regimens. A deeper mechanistic understanding of TAM-tumor crosstalk, paired with TAM-directed therapeutic innovations and empowered by artificial intelligence to decipher heterogeneity and forecast treatment responses, will help accelerate personalized medicine, bridging the gap between foundational research and clinical application to improve patient outcomes.

## Data Availability

Not applicable.
